# Studies on radiation sensitization efficacy by silymarin in colon carcinoma cells

**DOI:** 10.15190/d.2016.3

**Published:** 2016-04-01

**Authors:** Mitu Lal, Damodar Gupta

**Affiliations:** Division of Metabolic Cell Signaling and Research, Institute of Nuclear Medicine & Allied Sciences, DRDO, Brig SK Mazumdar Marg, Timarpur, Delhi, India

**Keywords:** silymarin, radiation sensitization, radiation, ROS, MMP

## Abstract

Recent reports demonstrated the role of silymarin as a cytoprotective agent for normal cells against ionizing or non-ionizing (UV) radiation, and in inhibiting the chemically initiated or promoted carcinogenesis in several malignancies, such as skin or prostate cancers. Silymarin is a plant flavonoid obtained from milk thistle; the main active principles in milk thistle are silybin (silibinin), sylichrisitin and silydianin, commonly referred as silymarin. In the present study, we aimed to investigate the radiation modulatory effects of silymarin on cancer cells. For this, we used the HCT-15 and RKO colon cancer cell lines as a model. Pre-irradiation treatment of cells with silymarin (20 mg/ml) followed by radiation exposure inhibits colon cancer cell proliferation and enhances cell death in a time-dependent manner. We have also examined the changes in p53 phosphorylation at Ser15, phosphorylation of p38 and their association with DNA damage. Silymarin was found to reduce proliferation of the human colon carcinoma cells in a concentration and time-dependent manner. Moreover, percentage of cell death was also increased in combined treatment (20µg/ml of silymarin + radiation). Our studies indicate that the combination increases the arrest of cells in G2/M phase of cell cycle, DNA damage-induced decrease in mitochondrial membrane potential (MMP) and a decrease of the reactive oxygen species (ROS) levels, which are associated with an increase in cell death. Altogether, these results suggest that silymarin sensitizes colon cancer cells to radiation, strategy with potential for colon cancer treatment. Noteworthy, since silymarin was previously shown to confer protection against radiation in at least some types of normal tissues, additional studies are needed to further investigate the potential of silymarin in colon cancer therapy when combined with radiation, its potential protective effects on normal tissues and its mechanisms of action.

## Introduction

Colon cancer is the third most commonly diagnosed cancer and the second leading cause of cancer death^1^. The established lifestyle risk factors for colorectal cancer include obesity, physical inactivity, smoking, a diet low in fruits, vegetables, fiber, processed meat etc. Approximately 95 percent of colorectal cancers are adenocarcinomas. Silymarin has already been evidentiated for its radioprotective efficacy both at *in vitro *and *in vivo levels*^2-5^*. *The anticancer effects of silymarin can be understood by various molecular mechanisms including blocking of carcinogenesis at different stages, such as initiation, promotion, and progression. Silymarin also known to possess anti-metastatic and anti-inflammatory activity and alter the balance between cell survival and apoptosis *via* expressions of cell cycle regulators and proteins involved in apoptosis^6-9^. Silymarin has also been known to possess anticancer efficacy and cause cell cycle arrest^10^. Silymarin induces apoptotic cell death *via* death receptor pathway. One of the major component of silymarin complex is silibin, apart from the other isomers, such as isosilibinin, silicristin, silidianin etc^7^.

Low linear energy transfer (LET) radiation is known to cause damage by inducing generation of reactive oxygen species (ROS). ROS plays an important role in cell signaling, intracellular redox status changes and cell death. It is evident that tumor suppressor gene p53 is induced by DNA damage^11^. It has been demonstrated that phosphorylation and dephosphorylation of some regulatory proteins play crucial role in controlling cell growth and apoptosis. Transcription factor like p53 can regulate various signal transduction pathways, including apoptosis. Mitogen activated protein kinase (MAPK) pathway consists of three tiered kinase (ERK, SAPK, and p38), involved in cell proliferation, differentiation and apoptosis ^12-14^.

Ionizing radiations are ubiquitous environmental agent, whose DNA-damaging effects are fairly well established. The comet assay permits detection of primary DNA damage and study of damage/ repair kinetics at the level of single cells ^15^. Activation of DNA damage sensors, transducers, cell cycle checkpoints have close association with damage-repair kinetics. This activation is known to arrest cells at a specific phase of the cell cycle, which may provide time to repair of damage and recovery of cells. Activation of the checkpoint is regulated by damage sensors, namely ATM and ATR ^11,16^. These kinases phosphorylate downstream targets in signal transduction cascade, eventually leading to cell cycle arrest. An important downstream target is p53, which plays a major role in apoptosis following DNA damage ^17,18^. In the present investigation, we studied the role of both p38 and p53, and their potential association with the DNA damage, mitochondrial physiology and ROS in relation to radiation sensitizing efficacy of silymarin in colon transformed cells (RKO and HCT-15).

## Materials and Methods

*Chemicals***. **All chemicals used in this study were of analytical grade and were either procured from Indian manufacturers (SRL India, HiMedia chemicals) or obtained from Sigma Aldrich, Thermo Scientific and Invitrogen (USA) and other companies. Minimum Essential Medium (EMEM); Roswell Park Memorial Institute-1640 (RPMI-1640), penicillin, streptomycin, trypsin, silymarin, protease and phosphatase inhibitors were procured from Sigma Chemicals (St. Louis, MO, USA), whereas fluorescent probes such as 3, 3’-DihexyloxacarbocyanineIodide [DiOC _6_ (3)], 5-(and-6)-chlormethyl2’,7’dichlorodihydrofluorescein diacetate acetyl ester [CM-H _2_DCFDA], propidium iodide (PI), sulphorhodamine-B (SRB), Foetal Bovine Serum (FBS) were procured from Invitrogen (USA).

*Cell cultures. *Colorectal adenocarcinoma (HCT-15) cells were obtained from National Centre for Cell Sciences, Pune, India and were maintained in RPMI-1640 medium, whereas RKO cells were maintained in Eagle’s Minimal Essential Medium (EMEM). Both media were supplemented with 10% (v/v) heat-inactivated FBS, 100 units/ml of penicillin and 100 µg/ml of streptomycin, pH 7.4 to maintain cells at 37°C in humidified atmosphere of 5% CO _2_: 95% air. All experiments were performed on exponentially growing cells and were subcultured twice a week as per requirement of each cell line.

*Preparation of silymarin solution. *Silymarin was dissolved in 90% RPMI and 10% ethanol v/v in media, under aseptic conditions. Treatments of cells with silymarin were performed as per indicated concentration(s), however cells were treated with silymarin 30 min prior to gamma radiation (2 Gy or 2.5 Gy) in the case of the combination.

*Gamma Irradiation of cells. *Irradiation was done using Bhabhatron-II Telecobalt unit (Bhabha Atomic Research Center, Mumbai, India) at 2.25-2.55Gy/min dose rate. Radiation dosimetry of unit was carried out by certified radiation safety officer in the institute. Baldwin Farmer secondary dosimeter and Fricke’s chemical dosimeter methodologies were also used.

*Treatment protocol. *HCT-15 cells were treated with different concentrations of Silymarin (ranging from 0.4 to 833µg/ml), to find out its nontoxic concentrations. In the case of radiation sensitization studies, cells were pretreated (30 min prior) with 20 µg/ml silymarin for both cell types (HCT-15 and RKO). RKO cells were treated with Silymarin, randing from 2.5 to 320µg/ml.

*Cytotoxicity studies using Sulforhodamine B assay*. Exponentially growing (3x10 ^3^) cells were seeded and incubated for proper attachment on surface (in 96 well plate) and thereafter cells were treated with increasing concentrations of silymarin (0.4 to 833µg/ml) using serial dilution process. After treatments, cells were washed with phosphate buffered saline, fixed using 10% TCA (protein precipitation; incubation 1h at 4°C) and washed with PBS to remove traces of TCA. Plates were air dried and cells were stained with 0.4% SRB in 1% acetic acid (w/v) in milli Q. After staining of cells, plates were washed 4-5 times with PBS to remove traces of unbound dye, kept inverted and air dried at room temperature. Finally, bound SRB were extracted using extraction buffer (10mM Tris base solution (w/v); pH 10.5). The absorbance of extracted dye was recorded by spectrophotometer (BIOTEK, USA) at λ565nm and λ690nm as reference wavelength ^19^.

*Clonogenic cell survival assay. *HCT-15 and RKO cells, were seeded ((250-300/ plate) in 60mm petridishes and allowed to attach for about 6-8h in CO _2 _incubator. For assessment of clonogenic efficacy plates were divided in four groups (control, silymarin alone, radiation alone and Silymarin + radiation), treated as per groups made and incubated for about 14-16 days (depending on their doubling time) to assess clonogenic efficacy or survival. After incubation, colonies were fixed and stained with crystal violet (0.1% in 70% methanol) ^20^. Colonies with more than 50 cells were counted and the plating efficiency (PE)/surviving fraction (SF) was determined. (PE= Colonies counted x 100/ cells plated; SF= no. of colonies formed/ (no of cells seeded x plating efficiency of control) x100%).

*Measurement of mitochondrial membrane potential (MMP). *Mitochondrial membrane potential was measured by flow cytometry (FACS-Caliber, Becton, Dickinson, USA) using 3, 3’-dihexyloxacarbocyanine iodide (DiOC _6 _(3); λ _ex_ 488nm, λ _em_ 530 nm). HCT-15 and RKO cells (1x10 ^6^cells) were plated in 60mm cell culture dishes and treated as mentioned in the treatment protocol section. Following various treatments, cells were washed with PBS and incubated with DiOC _6 _(3) (40 nM) at 37°C for 10min in the dark. Changes in MMP were acquired at least from 10,000 cells/ sample, previously described by Gupta et al ^21^.

*Measurement of Total ROS levels. *The changes in cellular ROS levels were measured using CM-H _2_DCFDA dye by flow cytometry, as described by Gupta et al.^21^. Briefly, 1x10^6 ^cells were plated in 60mm cell culture dishes for each condition, and after treatments, the cells were harvested and incubated with CM-H _2_DCFDA (10µM) at 37°C for 30min in dark. Following incubation, the fluorescence of oxidized dye was acquired at λ _ex_ 488nm *, *λ _em _max 530 nm from at least 10,000 cells/ sample. Results are expressed mean fluorescence.

*Cell cycle. *The changes in cell cycle phase distribution were measured by flow cytometry, using propidium iodide dye as described by Gupta et al ^20^. Briefly, following various treatments, cells were harvested, washed with PBS and fixed with ice cold 70% ethanol (24h at 4°C). After fixation, cells were centrifuged (1000rpm for 10min at room temperature) and washed with PBS twice to remove traces of ethanol. After washing, the cell pellet was suspended in PBS, containing RNAse (200µg/ml) and propedium iodide (50mg/ ml), and incubated for 30 min at 37°C. The fluorescence of PI was acquired by flow cytometry (λex 488nm and λem 636nm) from at least 10,000 cells.

*Comet assay. *Alkaline comet assays were performed to determine the amount of double strand DNA breaks, as described by Crosby et al.^17^. Briefly, treated cells were harvested by trypsinization and washed with PBS twice (1000g for 10 min at 4 °C). After washing cell pellet were suspended in low melting agarose (2% low melting agarose in PBS; w/v) and layered onto slides pre-coated with 1% agarose. The slides were moved on ice cold surface for gel formation and thereafter the cells were lysed for 40min at 4°C in lysis buffer. After lysis, DNA unwinding procedures were performed in alkaline conditions for 30min in the dark, at 4°C. This was followed by alkaline electrophoresis (1V/cm; for 30 min). After electrophoresis, slides were washed (with ice cold distilled water to remove traces of salts), dehydrated (using 70% ice cold ethanol) and air dried overnight in the dark. Comet results were acquired by staining of DNA with propidium iodide (5µg/ml) in the dark and acquired by fluorescence microscopy (Zoe, Fluorescent imager, Bio-Rad, USA).

*Western blotting. *Logarithmically growing cells were treated as described in the treatment protocol (20µg/ml silymarin and radiation or combination) and samples were collected at different time intervals. The western blotting procedure was performed as described by Gupta et al.^21^ with minor modifications. Briefly, following various treatments, cells were harvested and washed twice with ice cold PBS and thereafter, the cells were lysed in RIPA lysis buffer containing protease and phosphatase inhibitors (PMSF, sodium orthovanadate, protease and phosphatase inhibitor cocktail (Sigma Aldrich, USA), 0.5M sodium fluoride). For proper lysis, samples were vortexed on ice for 20min followed by centrifugation (12,000g, 4°C for 20min) and the supernatant was collected for western blotting and protein estimation. Protein concentration was measured by using BCA reagent (Sigma Aldrich, USA) and loaded in equal quantity (40-50µg protein/ well) for separation on 10% and 12% SDS-polyacrylamide gel. After gel electrophoresis, proteins were transferred onto PVDF membrane (Amersham, GE healthcare, Germany), using Tris-glycine transfer buffer containing 10% methanol. After transfer, membranes were blocked by using 4% BSA (in Tris-buffered saline containing tween 20 or TBST) or skimmed milk (5% in TBST) for 2h and thereafter the membranes were incubated overnight (at 4°C) with respective primary antibodies, phosphor-p53 (Ser15; 1:1000) phosphor-p38, (1:1000) and ß-actin (1:5000). The membranes were washed three times with TBST, to remove unbound and nonspecific primary antibody and incubated with appropriate secondary antibodies conjugated with horseradish peroxidase (HRP). The expression of proteins was measured by using the super signal West Pico chemiluminescent substrate (Thermo Scientific, USA).

*Data analyses and statistical evaluations. *Dose–response curves were produced using Prism 5.0, by using a Gaussian fit (GraphPad Software, San Diego, CA, USA), and the percentage survival was calculated using Student's t test from the graphical analysis. Fit comparison between survival curves was done with the F test. Changes in significance of ROS and MMP were analyzed by Student's t test, whereas for the combination ANOVA was used. For the graphical representation of the data, y-axis error bars representing ±SD are depicted and p values are shown at different levels of significance.

## Results

**Figure 1:**
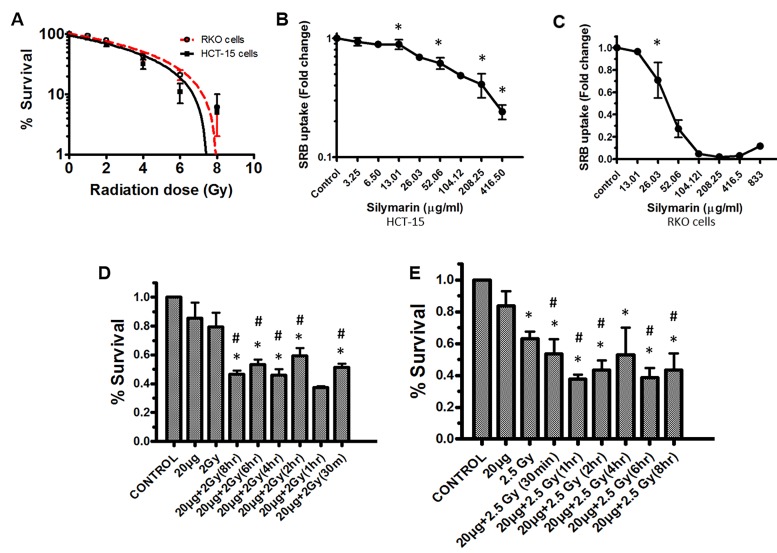
Changes in cell survival. **(A)** Radiation-mediated changes in the percentage survival of cells. Cells were exposed to increasing doses of radiation and clonogenic efficacy of RKO and HCT-15 cells was measured as described in the methods section. **(B and C)** Toxicity associated with increased concentrations of silymerin was measured by SRB uptake assay after 72h. **(D and E)** Effect of pre-radiation treatment with silymarin at different time intervals, on colony forming efficiency (CFE), in HCT-15 and RKO cells. After incubation (as described in the methods section) visible colonies were counted and surviving fraction was calculated with respect to control. Results are expressed as the percentage of cells surviving after treatment with respect to the control ±SD of three independent experiments. p < 0.05 was considered as level of significance (*Sham irradiated control vs radiation, #Radiation vs Combination).

*Cell survival and colony forming efficacy (CFE). *Cells were seeded and irradiated with increasing doses of ionizing radiation 0-8 Gy (**Figure 1** [A]). Exposure of cells to gamma radiation influenced CFE both of HCT-15 and RKO cells in dose dependent manner. Radiation dose in terms of lethality (LD _50_) were found to be 5Gy (for HCT-15) and 5.5Gy (for RKO cells) (**Figure 1** [A]). In the case of combination (silymarin; 20µg/ml + radiation; 2.5Gy for RKO and 2Gy for HCT-15) the treatment of cells significantly decreased survival with respect to radiation alone or sham irradiated control. The levels of sensitization were found to be relatively the same at all time points studied in the case of both RKO and HCT-15 cells (**Figure 1** [D, E]). 

*Sulforhodamine-B uptake assay. *Cells were seeded in 96 well plates and treated with increasing concentrations of silymarin for cytotoxicity studies. Treatment of cells with silymarin lowered cell proliferation in a concentration dependent manner. The 50% growth inhibition (GI _50_) for both HCT-15 and RKO cells was found to be at approximately 100µg/ml and 40µg/ml respectively (**Figure 1**[B]: HCT-15 cells and 1C: RKO cells). **

**Figure 2:**
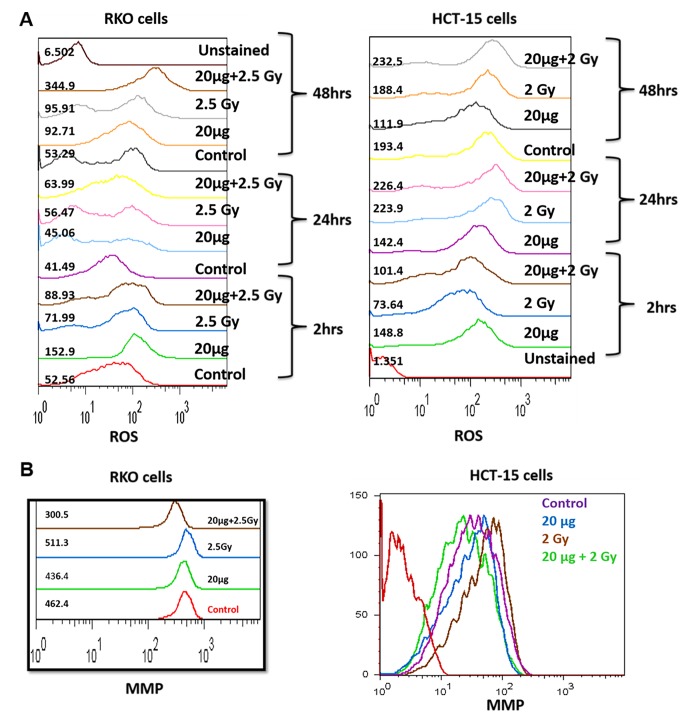
Changes in ROS generation and MMP **(A and B)** Effect of pre-irradiation treatment with silymarin at different time intervals on the generation of ROS and changes in MMP, in RKO and HCT-15 cells. After various treatments, ROS levels were measured fluorimetrically, by using CM-DCFH2DA or DiOC _6_(3) (Ex λ: 488 nm; Em λ: 530 nm; FL1 region) as described in materials and methods section. The mean fluorescence emission values are shown along with histograms.

*Reactive oxygen species levels.*Treatment of cells with silymarin followed by irradiation significantly increased generation of ROS in time-dependent manner, in the case of RKO cells, whereas the alterations in changes in ROS levels in the case of HCT-15 was found to be insignificant with respect to sham irradiated control or radiation alone, at all time points studied (**Figure 2** [A]). Exposure of RKO cells to 2.5Gy of gamma radiation significantly enhanced ROS levels with respect to sham irradiated control. Moreover, pre-irradiation treatment of cells with silymarin was found to enhance ROS levels over 6 fold, with respect to control, after 48h.

*Membrane potential changes.*Both RKO and HCT-15 cells treated with 20µg/ml of silymarin or exposed to radiation showed no alterations in MMP, as observed at 48h (**Figure 2** [B]). However, pre-irradiation treatment of both RKO and HCT15 cells showed significant decrease in MMP, suggesting the involvement of mitochondria in radiation sensitization.

**Figure 3:**
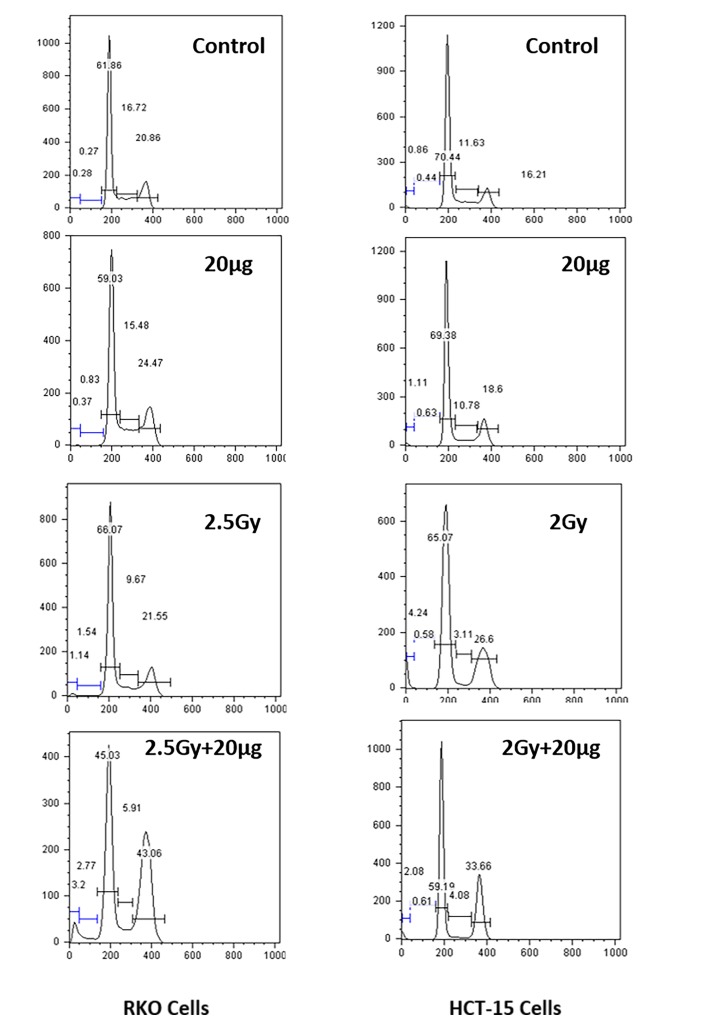
Studies on changes in Cell cycle phase distribution The changes in cell cycle phases were measured by using propidium iodide dye, as described in methodology. The results are represented as percentages of cells in specific phases of cell cycle.

*Effects on cell cycle phase distribution.*Alterations in distribution of cell cycle phases were assessed following various treatments (**Figure 3**). Treatment of HCT-15 / RKO cells with Silymarin showed no alteration in cell cycle phases observed at 72h, however cells exposed to radiation (2.5Gy for RKO and 2Gy for HCT-15) showed significant increase in G _2_/M population with respect to sham irradiated control. Silymarin treated RKO cells followed by radiation (2.5Gy) showed significant increase in G _2_/M population (43%) with respect to sham irradiated control (20%). Whereas in the case of HCT-15 cells the arrest at G _2_/M phase was found to be (34%) with respect to sham irradiated control (16%). 

**Figure 4:**
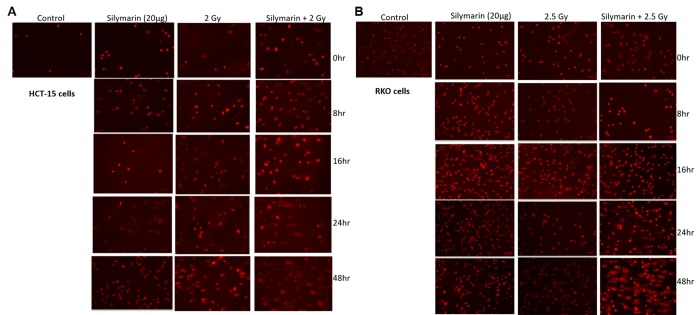
Detection of DNA damage by alkaline single cell electrophoresis (alkaline comet assay). Comet assays for HCT-15 cells **(A)** and RKO cells **(B)** were performed under alkaline conditions to determine the amount of double‑strand DNA breaks. Treated cells were collected, layered on slides, lysed and run into a horizontal elec­trophoresis chamber samples for ~30 min (1 V/cm at 4˚C). The slides were washed with deionized H _2_O to remove the alkaline buffer, dehydrated in 70% ice‑cold ethanol and air‑dried overnight. Slides were stained with PI (5 µg/ml) and examined by microscopy, as described in the methods section. Each experiment (performed three times) assessed radiation sensitization efficacy of silymarin specifically in relation to DNA damage.

*Detection of DNA damage by alkaline single cell electrophoresis (alkaline comet assay).*The Comet assay permits detection of primary DNA damage and study of repair kinetics at the level of single cells. We have measured the levels of DNA damage induced by ionizing radiation (IR) or its combination with silymarin (**Figure 4** [A, B]). Treatment of cells with silymarin showed no DNA damage at all time points studied (0, 8, 16, 24, 48 and 72h) with respect to sham irradiated control. The exposure of cells to radiation showed increased DNA damage in a time dependent manner, however cells were found to recover from DNA damage. The DNA damage was found to be significantly higher in the case of combination (silymarin+ radiation) at 0h time point, which was reduced at 8h in the case of both cell lines. However, the damage induced in the combined treatment was extensive and significantly higher in both cell lines. The increase in damage is time dependent, in the both cell lines.

**Figure 5:**
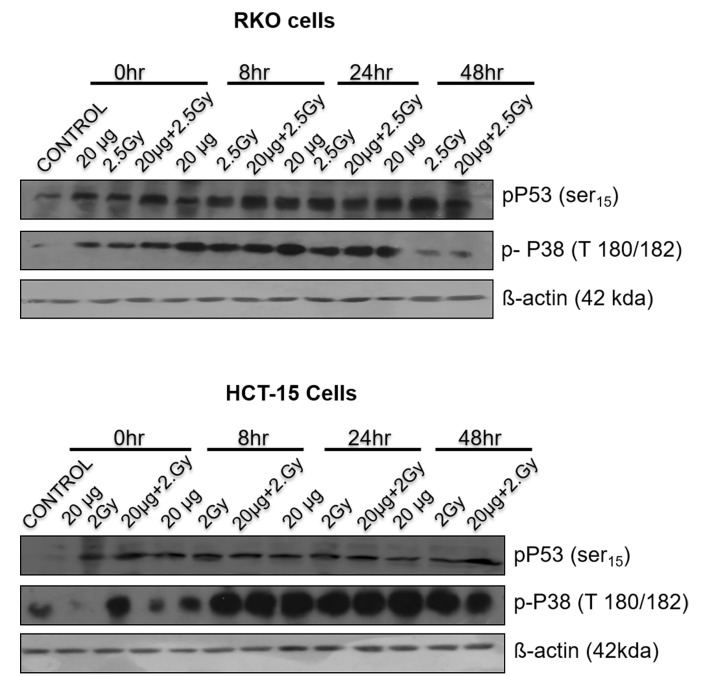
Role of p53 and p38 in silymarin induced radiation sensitization **.** Cells were treated, harvested (at different times) and samples were resolved on 10 or 12% SDS-PAGE, followed by the transfer on PVDF membranes and blotted using specific antibodies, as described in methods section. β-actin was used as a loading control.

*Phosphorylation of p53 and p38 proteins.*The change in phosphorylation status of p53 (Ser15) was measured following various treatments at different time intervals (0-48h), in both RKO and HCT-15 cells, by western blotting (**Figure 5**). Exposure of cells to radiation showed a time dependent increase of phospho-p53 levels for both RKO and HCT-15 cells. However, for combined treatment (silymarin+ radiation) the levels p53 phosphorylation were found to be significantly higher with respect to radiation in at all time points studied. **

It is well established that p38 MAPK plays a critical role in cell survival/ death, differentiation, apoptosis and metastasis. Radiation alone didn’t show activation of p38 (phosphor-p38) in RKO cells at 0h; however, combination treatment showed increase in activation of p38 at 0h time point and it was found to be higher till 8h. It showed similar levels thereafter, as compare to the radiation or sham irradiated control. In the case of HCT-15cells, the phospho-p38 was found to be higher in combined treatment with respect to radiation and sham irradiated group till 24h. Recent work has suggested a role for p38 MAPK in mediating radiation-induced pathways leading to cell apoptosis and growth inhibitory signals in cancer cells.

## Discussion

Ionizing radiation is known to cause damage to cellular biomolecules both directly by deposition of energy in them and indirectly by inducing ROS generation. Sudden burst in ROS can alter intracellular physiology in terms of redox status, gene expression and oxidative modification of biomolecules ^22,23^. These oxidative modifications in cellular biomolecules specifically in lipids, proteins, DNA etc., are very much dependent on the amount of dose absorbed by cells, antioxidant defense systems, cell cycle phase etc. Intrinsic antioxidant defense system, cellular energy levels and expression of anti-apoptotic proteins are known to support cells to cope with sudden changes in intracellular physiology or ROS levels. Various molecules like flavonoids, TLR agonists, antioxidant supplements, lignans, vitamins (A, D, E, C) are known for their radioprotective efficacy ^24-27^. However, treatment time, concentration, type of cell and others may determine fate of the cell.

In our previous studies we have identified radiation protection potential of silymarin ^2,4^. Silymarin is a cytoprotective agent for at least some types of normal cells against ionizing or non-ionizing (UV) radiation, and inhibits the chemically initiated or promoted carcinogenesis in several malignancies, such as skin or prostate cancers ^2,4,28,29^. Silymarin is extracted from the seeds and fruit of Silybum marianum (Compositae) and it is mixture of three structural isomeric components: silibinin, silydianine and silychristine. From a clinical application point of view, both silymarin and silibinin have been found to provide cytoprotection/hepatoprotection and radiation protection on normal cells. In the present study, we investigated the potential of silymarin in modulating radiation effects on colon carcinoma cells. The increase in concentrations of silymarin was associated with increased cytotoxicity and therefore we have utilized sub-lethal concentration of silymarin (20µg/ml) in combination with radiation 2Gy and 2.5Gy for both HCT-15 and RKO cells respectively (**Figure 1** [D, E]).

Exposure of cells to IRs is known to reduce cell survival; however, it varies form cell to cell, radiation dose, dose rate etc. RKO cells were found to be more resistant to IR as compared to HCT-15 (**Figure 1** [A]) and therefore, depending on radiation sensitivity, the selected radiation dose for HCT-15 and RKO for combination studies were 2 and 2.5 Gy. Pre-irradiation treatment of cells with silymarin (20µg/ml) followed by radiation exposure reduced proliferation of cells as measured by SRB uptake. These results are corroborated with increased ROS levels, a decrease in MMP, cell cycle arrest at G _2_/M phase and increased DNA damage. IR is known to enhance oxidative stress and thereby alter mitochondrial membrane potential in a time- and radiation-dose dependent manner. Over burden of oxidative stress leads to oxidative modification of cellular biomolecules, which ultimately causes cell death, if cells are not able to repair or recover from IR-induced stress. Pre-irradiation treatment of cells with silymarin showed enhanced ROS levels in a time-dependent manner. Mitochondria is known to consume over 85% of molecular oxygen during oxidative phosphorylation process and therefore it is a major site of oxidative stress. Moreover, availability of high amounts of unsaturated lipids and metal bound proteins makes them more vulnerable to oxidative stress ^20,25,30^. Oxidative modification of mitochondrial lipids and proteins is known to increase the leakage of electrons from electron transport chain, which further enhances superoxide radical generation and leads to a decrease in MMP. This thereby increases cell death. The decrease in MMP with time in pre-irradiation treatment of cells with silymarin could be due to the increase oxidative modification of biomolecules and opening of mitochondrial permeability transition pores (MPTP). Increased release of calcium ion from mitochondria is known to be associated with opening of MPTP, thereby activating various calmodulin proteins, such as endonucleases, lipases, proteases etc. The damage to DNA is known to activate p53 and MAPK pathways. In the present investigation, the cells treated with silymarin followed by radiation exposure showed an increase in DNA damage (comet assay), activated p53, p38 (as measured by phosphorylation status) levels and arrest of cells in G _2_/M phase after 48h, which suggest radiation sensitization efficacy of silymarin in colon cancer cells. However the mechanisms of cancer cells sensitization to radiation and the protective effect on normal cells remain to be further elucidated.

- **Silymarin is a cytoprotective agent for normal cells against ionizing or non-ionizing (UV) radiatino, and inhibits chemically-initiated or promoted carcinogenesis.**
- **OUR RESULTS demonstrate that silymarin sensitizes colon cancer cells to radiation, strategy with potential for colon cancer treatment.**
- **FUTURE EXPERIMENTS should further elucidate the mechanisms of silymarin-mediated sensitization of colon cancer cells to radiation and the potential protective effects on at least some types of normal tissues.**
